# Thank you

**DOI:** 10.1093/eurpub/ckab210

**Published:** 2022-02-01

**Authors:** Peter Allebeck, Diana Delnoij, Alastair Leyland, Walter Ricciardi, Róza Ádány

A scientific journal exists only because of the good will and assistance of its reviewers. We can only publish approximately a fifth of the papers we receive and, in order to publish really high-quality papers, we depend on the expertise of many people. During 2021 a total of 480 reviewers have helped us to decide about the papers that we should publish. Their names are listed below and we would like to take this opportunity to thank them. The list refers to reviewers completing their task until 1st of December 2021. 

Merita Berisha, Albania

Edward A. Ruiz-Narv´aez, United States

Mette Aadahl, Denmark

Mari Aaltonen, Finland

Muhammad Abbas, China

Michelle Addison, United Kingdom of Great Britain and Northern Ireland

Kees Ahaus, Netherlands

Marion Aichberger, Germany

Eva Aizpurua, United States

Yannis Alamanos, Greece

Tit Albreht, Slovenia

Djordje Alempijevic, Serbia

Denise Alexander, Ireland

Gianfranco Alicandro, Italy

Mirjam Allik, United Kingdom of Great Britain and Northern Ireland

Jordi Alonso, Spain

Carlos Alves, Portugal

Tarik Amer, United Kingdom of Great Britain and Northern Ireland

C. Ananth, United States

Ingelise Andersen, Denmark

Tatiana Andreeva, Ukraine

Peter Angelopoulos, Greece

Ahti Anttila, Finland

Cigdem Apaydin Kaya, Turkey

Franklin Apfel, United Kingdom of Great Britain and Northern Ireland

Ramiz Arabaci, Turkey

Nimmathota Arlappa, India

Hector Arolas, Spain

Lucia Artazcoz, Spain

Barbara Artnik, Slovenia

Hila Axelrad, Israel

Ali Babij, United States

Thomas Babor, United States

Eva Bacsne Baba, Hungary

Sekene Badiaga, France

Christine Baldwin, United Kingdom of Great Britain and Northern Ireland

Tom Baranowski, United States

Angelo Barbato, Italy

Liad Bareket-Bojmel, Israel

Pepita Barlow, United Kingdom of Great Britain and Northern Ireland

Orna Baron-Epel, Israel

Sarah Basharat, Pakistan

Alessandra Battisti, Italy

Cédric Baumann, France

Linda Bell, United Kingdom of Great Britain and Northern Ireland

Nadia Benahmed, Belgium

Fernando Benavides, Spain

Netta Bentur, Israel

Patrick Bergman, Sweden

Claudia Berlin, Germany

Marriot Bernadette, United States

Matthias Bethge, Germany

Maria Augusta, Bezerra, Brazil

Stuart Biddle, Australia

Éva Bíró, Hungary

Alexandre Bisdorff, Luxembourg

Ingunn Bjornsdottir, Denmark

Cecilia Björkelund, Sweden

Johan Bjørngaard, Norway

Jenni Blomgren, Finland

Kim Bloomfield, Denmark

Martin Bobak, United Kingdom of Great Britain and Northern Ireland

Stefania Boccia, Italy

Edwin Boezeman, Netherlands

Paolo Boffetta, Italy

Anette Ella, Boklund, Denmark

Anne Brabers, Netherlands

Henry Brandon, United States

Sven Bremberg, Sweden

Bronwyn Brew, Sweden

Ove Bring, Sweden

Óscar Brito Fernandes, Netherlands

Joanna Broad, New Zealand

Timothy Broady, Australia

Denise Brown, United Kingdom of Great Britain and Northern Ireland

Laura Brunelli, Italy

Martin Brussig, Germany

Bernd Brüggenjürgen, Germany

Richard Bränström, Sweden

Henrik Brønnum-Hansen, Denmark

Alex Burdorf, Netherlands

Jeni Burnette, United States

Bo Burström, Sweden

Susan Buskin, United States

Henrik Bøggild, Denmark

Chiara Cadeddu, Italy

Yutong Cai, United Kingdom of Great Britain and Northern Ireland

Tomaž Čakš, Slovenia

Adele Caldarella, Italy

Inês Campos-Matos, United Kingdom of Great Britain and Northern Ireland

Jaume Canela, Spain

Mayilee Canizares, Canada

Lorenzo Capasso, Italy

Simon Capewell, United Kingdom of Great Britain and Northern Ireland

Mabel Carabali, Canada

Martin Caraher, United Kingdom of Great Britain and Northern Ireland

Margarida Cardoso, Portugal

Margherita Caroli, Italy

Ewan Carr, United Kingdom of Great Britain and Northern Ireland

Elena Carrillo Álvarez, Spain

Dayan Carvalho Ramos Salles de Oliveira, Brazil

Jose Castillo, Spain

R. Catalano, United States

Nicolo Cavalli, United Kingdom of Great Britain and Northern Ireland

Jeremy Chancellor, United Kingdom of Great Britain and Northern Ireland

Justin Chapman, United States

Haohao Chen, China

Kong-Tong Chen, Taiwan

Lan Cheng, Germany

Palma Chillón, Spain

Maria-Dolores Chirlaque, Spain

Eun Choi, United States

Anne Christensen, Denmark

Nain-Feng Chu, Taiwan

Bronwyn Clark, Australia

Miriam Colombo, United States

Giuseppe Costa, Italy

Claudia Costa, Portugal

Jen Coury, United States

Matty Crone, Netherlands

Paolo Crosignani, Italy

Yuehua Cui, China

Sarah Cuschieri, Malta

Bruno da Costa, Brazil

Espen Dahl, Norway

Anna-Karin Danielsson, Sweden

Zuzana Dankulincova, Slovakia

Jean-Francois Daoust, United Kingdom of Great Britain and Northern Ireland

Rituparna Das, India

Mike Daube, Australia

Barbara D'Avanzo, Italy

A. Davis, United Kingdom of Great Britain and Northern Ireland

Guy De Backer, Belgium

Sascha de Breij, Netherlands

Astrid de Wind, Netherlands

Patricia M. Dekkers-Sánchez, Netherlands

Annarosa del Mistro, Italy

Lanfranco D'Elia, Italy

Michael Deml, Switzerland

Evangelia Demou, United Kingdom of Great Britain and Northern Ireland

Zelalem Destaw, Ethiopia

Giorgio Di Gessa, United Kingdom of Great Britain and Northern Ireland

Giuseppe Di Martino, Italy

Sara Dias, Portugal

Julio Diaz, Spain

Finn Diderichsen, Denmark

Jennifer Dixon, United Kingdom of Great Britain and Northern Ireland

Felıcitas Domınguez-Berjon, Spain

Yanghui Dong, China

Danny Dorling, United Kingdom of Great Britain and Northern Ireland

Gerardine Doyle, Ireland

Dunja Dreesens, Netherlands

Sven Drefahl, Sweden

Gertjan Driessen, Netherlands

Katarzyna Dubas-Jakóbczyk, Poland

Nathalie Duchange, France

Xavier Duran, Spain

Magdalena Durda-Masny, Poland

Raika Durusoy, Turkey

Marion Duval, France

Hans Duvekot, Netherlands

Christina Edwards, Norway

Frida Eek, Sweden

Boris Ehrenstein, Germany

Peter Eibich, Germany

Marion Eisele, Germany

Mikael Ekblad, Finland

Anna Mia Ekström, Sweden

Nuha El Sharif, Palestine, State of Eric Emerson, United Kingdom of Great Britain and Northern Ireland

Cornelia Enzenbach, Germany

Saffet Erdoğan, Turkey

Erdem Erkoyun, Turkey

Sofija Ernesta, Australia

John Fanourgiakis, Greece

Alan Farrier, United Kingdom of Great Britain and Northern Ireland

Paula Feder-Bubis, Israel

Gracia Fellmeth, United Kingdom of Great Britain and Northern Ireland

Joao Ferreira-Pinto, United States

Wiesław Fidecki, Poland

Florian Fischer, Germany

Claudia Fischer, Netherlands

Patricia Fitzpatrick, Ireland

Maria Elena Flacco, Italy

Maria Fleischmann, Netherlands

Pär Flodin, Sweden

Mari-Ann Flyvholm, Denmark

Anders Foldspang, Denmark

Carla Fornari, Italy

Emma Fransson, Sweden

Inês Fronteira, Portugal

Thomas Fröhlich, Germany

Sari Fröjd, Finland

Judith Fuchs, Germany

Sonsoles Fuentes, Spain

Silvano Gallus, Italy

Mª Victoria Galvez, Spain

Thomas Gamsjäger, Austria

Kristin Ganahl, Austria

Inmaculada García Martínez, Spain

Heike Garritsen, Netherlands

Tony Gatrell, United Kingdom of Great Britain and Northern Ireland

Vijay Gc, United Kingdom of Great Britain and Northern Ireland

Laurence Geebelen, Belgium

Delaram Ghodsi, Iran

Monica Giancotti, Italy

Theodoros Giannouchos, United States

Katja Gillander Gådin, Sweden

Felix Gille, Switzerland

Alejandro Gil-Salmerón, United Kingdom of Great Britain and Northern Ireland

Paolo Giorgi Rossi, Italy

Roberto Gnavi, Italy

Pere Godoy, Spain

Rosa Gofin, Israel

Bernard Gonik, United States

Angelina Gonzalez Viana, Spain

Francisco-Javier Gonzalez-Barcala, Spain

Nick Goodwin, United Kingdom of Great Britain and Northern Ireland

Unni Gopinathan, Norway

Giuseppe Gorini, United States

Igor Grabovac, Austria

Guendalina Graffigna, Italy

Irina Grafova, United States

Eleanor Grieve, United Kingdom of Great Britain and Northern Ireland

Anne Griffin, Ireland

Eva Grill, Germany

Peter Groenewegen, Netherlands

Laurence Gruer, United Kingdom of Great Britain and Northern Ireland

Maria Gualano, Italy

Pedro Gullon, Spain

Maria Guzman-Castillo, Finland

Isabel Hach, Germany

Faisal Hakeem, United Kingdom of Great Britain and Northern Ireland

Torleif Halkjelsvik, Norway

Virginie Halley des Fontaines, France

Liping Hao, China

Beatrijs Haverkamp, Netherlands

Louise Hawkley, United States

Samah Hayek, Israel

Kène Henkens, Netherlands

Gunnel Hensing, Sweden

Anders Hjern, Sweden

Sarah Hoeck, Belgium

Theodore Holford, United States

Christine Holmberg, Germany

C. Homer, Australia

H. Hoven, Germany

Chang-Ming Hsieh, United States

Min Hu, China

Kuo-Cherh Huang, Taiwan

Juliette Hussey, Ireland

Lena Hünefeld, Germany

M. Ishikane, Japan

Nazrul Islam, United Kingdom of Great Britain and Northern Ireland

Rachel Jackson-Leach, United Kingdom of Great Britain and Northern Ireland

Teresa Janevic, United States

Janko Jankovic, Serbia

Domantas Jasilionis, Germany

Melanie Jones, United Kingdom of Great Britain and Northern Ireland

Joshua Winston Joseph, United States

Christian Jung, Germany

Karsten Jørgensen, Denmark

M. Kakizaki, Japan

Rainer Kaluscha, Germany

Daiga Kamerāde, United Kingdom of Great Britain and Northern Ireland

Samuli Kangaslampi, Finland

Brystana Kaufman, United States

Lars Kayser, Denmark

Carl Kendall, United States

Erin Kenzie, United States

Evert Ketting, Netherlands

Myriam Khlat, France

Katarzyna Kissimova-Skarbek, Poland

Niek Klazinga, Netherlands

A.K. Knudsen, Norway

Tetsuro Kobayashi, Japan

Stefan Kohler, Germany

Natasja Koitzsch Jensen, Denmark

Naoru Koizumi, United States

Margarita Kokkorou, United Kingdom of Great Britain and Northern Ireland

Rudolf Kool, Netherlands

Karolina Kósa, Hungary

Karoline Kragelund Nielsen, Denmark

Margareta Kristenson, Sweden

Alfgeir Kristjansson, Iceland

Tove Kristoffersen, Norway

Anton Kunst, Netherlands

Oliver Kurzai, Germany

Y. Kusama, Japan

Thomas Kühlein, Germany

Carlo La Vecchia, Italy

Jeremy Labrecque, Netherlands

Nathalie Lahoud, Lebanon

Taavi Lai, Estonia

Tea Lallukka, Finland

Giovanni Lamura, Italy

Peter Lansberg, Netherlands

Olga Laosa, Spain

Andrea Lavazza, Italy

Stephen Lawoko, Sweden

Teresa Leao, Portugal

Wonkyong Lee, Canada

Seo Yoon Lee, Korea, the Republic of Hans Lehrach, Germany

Lijian Lei, China

Liliya Leopold, Netherlands

Peter Lepping, United Kingdom of Great Britain and Northern Ireland

Jie Li, United States

Yige Li, United States

Yajun Liang, Sweden

Laura Lindberg, Denmark

Hedda Lippus, Estonia

Emily Long, United Kingdom of Great Britain and Northern Ireland

Francesco Longo, Italy

E. Lothian, United Kingdom of Great Britain and Northern Ireland

Rikke Lund, Denmark

Ulf Lundberg, Sweden

Torkild Lyngstad, Norway

Mhairi Mackenzie, United Kingdom of Great Britain and Northern Ireland

Ignacio Madero-Cabib, Chile

Manfred Maier, Austria

Elena Manescu, Romania

M. Marti, Spain

Leslie Martin, United States

Jolanda Mathijssen, Netherlands

T. Matsubayashi, Japan

Xián Mayo, Spain

Joanna Mazur, Poland

Jim McCambridge, United Kingdom of Great Britain and Northern Ireland

Mary McEniry, United States

Philip McLoone, United Kingdom of Great Britain and Northern Ireland

Erik Melén, Sweden

Aziz Mensah, Germany

Emmanouil Mentzakis, United Kingdom of Great Britain and Northern Ireland

Gwenn Menvielle, France

Christian Meyer, Germany

Qun Miao, Canada

Dusanka Micetic-Turk, Slovenia

Reyaj Mikrani, China

Pilar Modamio, Spain

S. Modrek, United States

Susanne Moebus, Germany

Anders Moellekaer, Denmark

Bishav Mohan, India

Jill-Marit Moholt, Norway

Anu Molarius, Sweden

Linda Montanari, Portugal

Simon Moore, United Kingdom of Great Britain and Northern Ireland

Britt Morthorst, Denmark

Shayan Mostafaei, Iran

Maria Mourão-Carvalhal, Portugal

Massimo Mucciardi, Italy

Madhuri Mulekar, United States

Yuichi Muraki, Japan

Akinori Nakata, Japan

Cathrine Nakken, Denmark

Elin Naurin, Sweden

Luciana Neamtiu, Italy

Davide Negrini, Italy

Georgios Nikolopoulos, Cyprus

Peter Nilsson, Sweden

Thor Norström, Sweden

Leslie Ogilvie, Germany

Atte Oksanen, Finland

Anna Oksuzyan, Germany

Valérie Olié, France

Jacques Orvain, France

Andrea Padoan, Italy

Demosthenes Panagiotakos, Greece

Kelly Parsons, United Kingdom of Great Britain and Northern Ireland

Anna Pearce, United Kingdom of Great Britain and Northern Ireland

Sergio Perelman, Belgium

Julian Perelman, Portugal

Pamela Pereyra-Zamora, Spain

Ivan Perry, Ireland

Armando Peruga, Switzerland

T. Petitte, United States

Paola Piccolo, Italy

Sylvain Pichetti, France

Matthias Pierce, United Kingdom of Great Britain and Northern Ireland

Peter Piko, Hungary

Stefan Pilcher, Switzerland

Paul Pinsky, United States

Sami Pirkola, Finland

Prisco Piscitelli, Italy

Carme Planas-Campmany, Spain

Sabine Plancoulaine, France

Thomas Plümper, Austria

Andrea Poscia, Italy

Alejandro Prieto Ayuso, Spain

Tina Purnat, Sweden

Ossi Rahkonen, Finland

Francesco Ramponi, Spain

Sarah Reed, United Kingdom of Great Britain and Northern Ireland

Sijmen Reijneveld, Netherlands

Sofia Ribeiro, Portugal

Margret Rihs, Switzerland

Arja Rimpelä, Finland

Kaaja Risto, United States

Renne Rodrigues, Brazil

Fernando Rodriguez-Artalejo, Spain

Vladimir Rogalewicz, Czech Republic

Manuel Romero-Gomez, Spain

Jérôme Ronchetti, France

Sam Rowlands, United Kingdom of Great Britain and Northern Ireland

Sarah Rozenblum, United States

Reiner Rugulies, Denmark

Abigail Emma Russell, United Kingdom of Great Britain and Northern Ireland

Keith Sabin, Switzerland

Luís Saboga Nunes, Portugal

Muhammad Saeed, Pakistan

Edgar Samarasundera, United Kingdom of Great Britain and Northern Ireland

Laura Samuel, United States

João Santos, Portugal

Tugce Schmitt, Netherlands

Claudia Schoenborn, Belgium

Naomi Schwartz, Canada

David Seal, United States

Chris Segrin, United States

Vida Sheikh, United States

Wensong Shen, Hong Kong

Mary Shilalukey Ngoma, Zambia

Colin Shore, United Kingdom of Great Britain and Northern Ireland

Karri Silventoinen, Finland

Erika Siu, United States

Sara Souza, Canada

Pinar Soysal, Turkey

Jacob Spallek, Germany

Domonic Sparkes, United Kingdom of Great Britain and Northern Ireland

Adina Stan, Romania

Denes Stefler, United Kingdom of Great Britain and Northern Ireland

Robert Stein, United States

Timo Ståhl, Finland

Harry Sumnall, United Kingdom of Great Britain and Northern Ireland

Alma Sörberg Wallin, Sweden

Jennifer Taber, United Kingdom of Great Britain and Northern Ireland

M. Tallarek, Germany

David Taylor-Robinson, United Kingdom of Great Britain and Northern Ireland

Liesbeth Temme, Netherlands

Jürgen Thelen, Germany

Ioannis Theodossiou, United Kingdom of Great Britain and Northern Ireland

Betsy Thom, United Kingdom of Great Britain and Northern Ireland

Siri Thor, United States

Maija Toivakka, Finland

Hanna Tolonen, Finland

P. Tyrer, United Kingdom of Great Britain and Northern Ireland

Sven Törnberg, Sweden

Emre Umucu, United States

Antonis Valachis, Greece

Maria Valenzuela, United Kingdom of Great Britain and Northern Ireland

David Walsh, United Kingdom of Great Britain and Northern Ireland

J. van der Velden, Netherlands

Hans van der Wouden, Netherlands

Frank van Lenthe, Netherlands

Hanna van Solinge, Netherlands

Irene van Valkengoed, Netherlands

Carine Vande Voorde, Belgium

P. Vanella, Germany

Rakhi Vashishtha, Australia

H. Gilbert Welch, United States

Jacques Wels, Belgium

Uno Wennergren, Sweden

Robert Verheij, Netherlands

Margaret Whitehead, United Kingdom of Great Britain and Northern Ireland

Francesco Violante, Italy

Marianna Virtanen, Sweden

Dirk Witteveen, United Kingdom of Great Britain and Northern Ireland

Alejandra Vives, Chile

Sabine Vogler, Austria

G Zagury, China

Jennifer Zeitlin, France

Caixia Zhang, China

Dong Zhang, United States

Pengxiang Zhao, Sweden

Bin Zhou, United Kingdom of Great Britain and Northern Ireland

Corina-Aurelia Zugravu, Romania

Gunnar Ågren, Sweden

Johan Åhlén, Sweden

Per-Olof Östergren, Sweden

Hilal Özcebe, Turkey

